# Gastric cancer prevention by *H. pylori* eradication in China: a meta-analysis of 8 high-quality RCTs in targeted screening populations

**DOI:** 10.3389/fonc.2026.1789299

**Published:** 2026-04-01

**Authors:** Junpeng Liao, Jian Sun, Tingting Jin, Jiashun Li, Deyu Li, Hailong Wang

**Affiliations:** Department of Gastroenterology, Sixth Affiliated Hospital of Xinjiang Medical University, Sixth Affiliated Hospital of Xinjiang Medical University, Ürümqi, China

**Keywords:** China, gastric cancer, *Helicobacter pylori* eradication, meta-analysis, targeted screening

## Abstract

**Objective:**

Currently, existing studies lack specific long-term follow-up subgroup analyses for the Chinese population and fail to clarify how sample size and recruitment strategies affect the heterogeneous efficacy of Hp eradication. We therefore integrated data from Hp eradication therapy studies in Chinese Hp-positive patients with ≥3 years of follow-up, quantitatively analyzed the therapy’s impact on gastric cancer risk, and explored preventive effect differences across subgroups with varying sample sizes. This study aims to provide evidence-based support for clinical decision-making in gastric cancer primary prevention and the formulation of public health strategies.

**Methods:**

We searched six major databases: PubMed, Embase, the Cochrane Library, Web of Science, Wanfang Database, and CNKI (China National Knowledge Infrastructure). The search included all relevant randomized controlled trials (RCTs) from each database’s inception to December 31, 2025. After screening based on inclusion and exclusion criteria, we used RevMan 5.3 software for risk of bias assessment, effect size pooling, and subgroup analysis. We assessed evidence quality using the GRADE approach.

**Results:**

A total of 8 high-quality randomized controlled trials (RCTs) were included, with 260,609 participants (118,425 receiving Helicobacter pylori [Hp] eradication therapy). Pooled analysis showed that Hp eradication therapy significantly reduced gastric cancer risk by 18% (RR = 0.82, 95%CI: 0.73–0.91, P = 0.0004). Sample size subgroup analysis revealed that the conventional sample mixed group had a 45% lower gastric cancer risk (RR = 0.55), significantly outperforming the ultra-large sample community group (RR = 0.88). For follow-up duration, the subgroup with ≥10 years of follow-up showed a 47% reduction in gastric cancer risk (RR = 0.53, P<0.0001), while short-term intervention (≤5 years) showed a trend toward reduced risk without reaching statistical significance.

**Conclusion:**

Helicobacter pylori (Hp) eradication therapy significantly reduces gastric cancer risk in Chinese Hp-positive individuals. It yields more pronounced benefits when participants are recruited via small- and medium-sized community screening or clinical channels, with clear gains observed in long-term follow-up (≥10 years). We recommend promoting this therapy among clinical high-risk populations and in precise community screening to enhance intervention cost-effectiveness.

**Systematic Review Registration:**

https://www.crd.york.ac.uk/prospero/display_record.php?ID=CRD420261277174, identifier CRD420261277174

## 1 Introduction

Helicobacter pylori (Hp) infection is a common chronic infectious disease closely related to the occurrence of gastric cancer. It is a key risk factor that causes chronic inflammation of the gastric mucosa, promotes the progression of precancerous lesions, and thereby increases the risk of gastric cancer (1), which is usually caused by long-term uncontrolled infection. Epidemiological studies have shown that the Hp infection rate in China is as high as 40%–45% (2), and new gastric cancer cases in China account for 50% of the global total each year, ranking first in the world in both number and proportion (3), which significantly threatens life and health and increases the social medical burden (1) (2).

Hp eradication therapy is considered an important measure to reduce the risk of gastric cancer, and most studies have shown that standardized eradication therapy can reduce gastric mucosal injury and delay the progression of lesions (4) (5) (6). Without timely and effective eradication therapy at an early stage, the risk of gastric cancer will increase significantly, and the risk of progression to refractory precancerous lesions or even gastric cancer will rise (7) (8). However, controversies and deficiencies still exist in studies on the long-term preventive effect of Hp eradication therapy against gastric cancer in Chinese Hp-positive patients. Some studies are unable to accurately reflect the long-term impact of eradication therapy on gastric cancer due to the short follow-up duration (9) (10); several previous systematic reviews and Meta-analyses have not focused on the specificity of the Chinese population and lacked targeted analyses of subgroups with different sample sizes (11) (12), which may be due to insufficient consideration of the differences in Hp infection characteristics, diagnosis and treatment patterns, and gastric cancer risk among different Chinese populations (13).

Many clinical studies and evidence-based medical analyses on Helicobacter pylori (Hp) eradication therapy have been conducted, including comparisons of efficacy among different treatment regimens and assessments of short-term prognosis (4) (14). However, there is still a lack of systematic and comprehensive analysis on the effect of Hp eradication therapy on the risk of gastric cancer in Chinese Hp-positive patients with follow-up ≥ 3 years. In particular, the differences in the preventive effect of Hp eradication therapy against gastric cancer among subgroups with different sample sizes have not been clarified. Therefore, this systematic review and meta-analysis takes Chinese Hp-positive patients with follow-up ≥ 3 years as the study population. We systematically searched and integrated relevant research data, quantitatively analyzed the effect of Hp eradication therapy on the risk of gastric cancer, and focused on exploring the differences in its preventive effect against gastric cancer in subgroups with different sample sizes. This study aims to provide evidence-based evidence for clinical decision-making and public health strategy formulation in the primary prevention of gastric cancer in Chinese Hp-positive patients.

## 2 Methods

### 2.1 Search strategy

We searched Chinese and English literature across six databases: PubMed, the Cochrane Library, Embase, Web of Science, CNKI (China National Knowledge Infrastructure), and Wanfang. Both subject headings and free words were used for indexing, with keywords including “gastric cancer”, “Helicobacter pylori”, “eradication”, “randomized”, et al. We systematically retrieved randomized controlled trials (RCTs) on gastric cancer incidence after H. pylori eradication therapy, covering all records from each database’s inception to December 31, 2025. Grey literature (e.g., conference materials, academic theses) was not excluded during database searches. Taking PubMed as an example, the search strategy is shown in **Table 1**, with complete strategies for all databases provided in **Supplementary File 1**. To minimize bias and enhance search transparency, we manually screened references of the finally included studies and high-quality systematic reviews/meta-analyses related to H. pylori eradication and gastric cancer prevention, avoiding missing potential eligible literature. Only Chinese and English articles were included. We also systematically searched grey literature per the study’s inclusion/exclusion criteria, but no eligible grey literature was identified, so none was incorporated. This entire process was collectively verified by the research team.

**Table 1:** Search strategy: PubMed example.

#1	(Stomach Neoplasms [Mesh]) OR (Neoplasm, Stomach[Title/Abstract] OR Stomach Neoplasm[Title/Abstract] OR Gastric Neoplasms[Title/Abstract] OR Gastric Neoplasm[Title/Abstract] OR Neoplasm, Gastric[Title/Abstract] OR Neoplasms, Gastric[Title/Abstract] OR Neoplasms, Stomach[Title/Abstract] OR Cancer of Stomach[Title/Abstract] OR Stomach Cancers[Title/Abstract] OR Cancer of the Stomach[Title/Abstract] OR Gastric Cancer[Title/Abstract] OR Cancer, Gastric[Title/Abstract] OR Cancers, Gastric[Title/Abstract] OR Gastric Cancers[Title/Abstract] OR Stomach Cancer[Title/Abstract] OR Cancers, Stomach[Title/Abstract] OR Cancer, Stomach[Title/Abstract] OR Gastric Cancer, Familial Diffuse[Title/Abstract])
#2	(Helicobacter pylori [Mesh]) OR (Campylobacter pylori subsp. pylori[Title/Abstract] OR Campylobacter pyloridis[Title/Abstract] OR Campylobacter pylori[Title/Abstract] OR Helicobacter nemestrinae[Title/Abstract] OR HP[Title/Abstract])
#3	((Disease Eradication [Mesh])) OR (Disease Eradications[Title/Abstract] OR Eradication, Disease[Title/Abstract] OR Eradications, Disease[Title/Abstract] OR Disease Elimination[Title/Abstract] OR Disease Eliminations[Title/Abstract] OR Elimination, Disease[Title/Abstract] OR Eliminations, Disease[Title/Abstract] OR Elimination*[Title/Abstract] OR Eradication*[Title/Abstract])
#4	((((((Randomized Controlled Trials as Topic[Mesh]) OR (Randomized controlled trial[Title/Abstract])) OR (Clinical Trials, Randomized[Title/Abstract])) OR (Trials, Randomized Clinical[Title/Abstract])) OR (Controlled Clinical Trials, Randomized[Title/Abstract])) OR (RCT[Title/Abstract])) OR (Randomized[Title/Abstract])
#5	#1AND#2AND#3AND#4

### 2.2 Inclusion and exclusion criteria

A follow-up duration of ≥3 years was set as one of the core inclusion criteria in this study. This threshold is consistent with the core concept of the Guideline for Gastric Cancer Screening and Early Diagnosis and Treatment (2024 Edition) (15). This guideline clearly states that the follow-up period for high-risk gastric cancer populations should exceed 3 years to evaluate intervention effects. It also matches the time-dependent process from chronic gastric mucosal inflammation and precancerous lesions caused by Hp infection to gastric cancer. A 3-year follow-up is sufficient to capture the cancer-preventive effect of eradication therapy and avoid underestimating its value due to too short follow-up. Meanwhile, this study focused on the direct association between Hp eradication therapy and new gastric cancer cases. The primary outcome was newly diagnosed gastric cancer during follow-up. Epidemiological models related to gastric cancer and antibody indicators such as CAG-a were not considered in the inclusion and exclusion criteria.

#### 2.2.1 Inclusion criteria

(1) randomized controlled trial (RCT) studies published in Chinese or English; (2) Chinese study population; (3) follow-up duration of ≥3 years with complete follow-up data; (4) participants being patients diagnosed with Helicobacter pylori (Hp) infection via urea breath test, pathological biopsy, serological test or other methods; (5) the intervention group receiving recognized Hp eradication regimens (including but not limited to proton pump inhibitor [PPI]-based triple therapy, bismuth-containing quadruple therapy, H2-receptor antagonist triple therapy) with an eradication rate of ≥50%; (6) the control group receiving placebo or no Hp eradication treatment; (7) newly diagnosed gastric cancer during the follow-up period as the outcome measure.

#### 2.2.2 Exclusion criteria

(1) Follow-up duration of < 3 years or incomplete follow-up data; (2) Enrollment of patients with confirmed gastric cancer, severe precancerous lesions, a history of gastric surgery, or severe underlying diseases (e.g., severe hepatic and renal dysfunction); (3) Studies that failed to clearly report participants’ basic characteristics; (4) Identical Hp eradication interventions in both the intervention and control groups (either both receiving or neither receiving the therapy) or no clear distinction between the two groups’ interventions; (5) Unreported gastric cancer incidence or gastric cancer diagnoses lacking histopathological evidence.

### 2.3 Literature screening and data extraction

This study strictly implemented independent and repeated procedures for literature screening and data extraction throughout the whole process. Two researchers independently conducted preliminary screening by checking the titles and abstracts of literatures, and excluded duplicate, irrelevant and review articles. Then they carefully read the remaining literatures for further screening and finally selected eligible literatures. The whole process was repeated by a third researcher. When the two researchers had disagreements, they discussed with the third researcher to confirm the final eligible literatures, and a flow chart was drawn using the PRISMA Flow diagram (16). Before data extraction, the research team made a standardized data extraction form in advance, and performed pre-extraction on 2 randomly selected literatures to optimize the form items, so as to ensure the integrity and consistency of the extracted information. Then two researchers independently extracted data from eligible literatures with the optimized form, and cross-checked the data after extraction. Items with inconsistent data were rechecked with the original texts. If disagreements still existed, a third researcher made the final decision. We extracted the following information from eligible literatures: first author, publication year, country where the trial was carried out, sample size, person-years, mean age, baseline status, smoking and drinking status of the study population, intervention measures, control measures, eradication therapy regimen, eradication rate, eradication control method, follow-up method, follow-up interval, follow-up duration and outcome indicators (**Tables 2**, **3**).

**Table 2:** Information extraction.

No.	Study	Country	Disease at baseline	Enrolled	Person-years	Baseline characteristic	Intervention		Regimen of eradication therapy	Eradication rate(%)	Follow-up periods (yr)
				Exp/Ctr	Exp/Ctr		Exp	Ctr			Exp/Ctr
1	Yi-Chia Lee 2024 (18)	Taiwan, China	Non-gastric cancer	63508/88995	350575/480573	50-69 years, Non-gastric cancer	Hp eradication	placebo	1st: ESZ(40)/AMPC(1000), BID 5D; 2nd: ESZ(40)/CAM(500)/MNZ(500), BID 5D; Salv: ESZ(40)/AMPC(1000)/LVX(500), QD/BID 10D	91.90%	5.7/5.4
2	Kai-Feng Pan 2024 (8)	Shandong, China	Non-gastric cancer	52026/50304	607834/587709	Hp-infected, Non-gastric cancer	Hp eradication	placebo	OPZ(20)/TCY(750)/MNZ(400)/BKC(300), BID/TID 10D	72.90%	11.8
3	Lingjun Yan 2022 (10)	Fujian, China	Non-gastric cancer	817/813	18340/17656	Hp-infected,35–65 years, Non-gastric cancer	Hp eradication	placebo	1st: OPZ(20)/AMPC-CLA(750)/MNZ(400), BID 10D; Salv: Quadruple therapy, 7D (drugs unreported)	83.70%	26.5
4	Wen-Qing Li 2019 (19)	Shandong, China	Non-gastric cancer	1130/1128	25199/25154	Hp-infected,35–64 years, Non-gastric cancer	Hp eradication	placebo	AMPC(1000)/OPZ(20), BID 14D; Salv: Same regimen, 14D	Not reported	22.3
5	Zhou Liya 2014 (20)	Shandong, China	Non-gastric cancer	276/276	2760/2760	Hp-infected,35–75 years, Non-gastric cancer	Hp eradication	placebo	OPZ(20)/AMPC(1000)/CAM(500), BID 7D	88.89%	10
6	Tang 2010 (21)	Jiangsu, China	Non-gastric cancer	118/118	354/354	Hp-infected,30–70 years, Non-gastric cancer	Hp eradication	placebo	1st: OPZ(20)/CAM(500)/AMPC(1000), BID 7D; Salv: BKC-containing quadruple therapy (drugs unreported)	82.10%	3
7	W K Leung 2004 (22)	Shandong, China	Non-gastric cancer	295/292	1475/1460	Hp-infected,52.0 ± 8.1 years, Non-gastric cancer	Hp eradication	placebo	OPZ(20)/AMPC(1000)/CAM(500), BID 7D	74.50%	5
8	Benjamin C Y Wong 2012 (23)	Shandong, China	Non-gastric cancer	255/258	1220/1230	Hp-infected,35-64 years, Non-gastric cancer	Hp eradication	placebo	OPZ(20)/AMPC(1000)/CAM(500), BID 7D	69.50%	5

Non-gastric cancer: patients had no history of gastric cancer, but were unknown to have peptic ulcer and dysplasia; ESZ, Esomeprazole; OPZ, Omeprazole; AMPC, Amoxicillin; CAM, Clarithromycin; MNZ, Metronidazole; LVX, Levofloxacin; TCY, Tetracycline; BKC, Bismuth potassium citrate; AMPC-CLA, Amoxicillin-clavulanate; Salv, Salvage therapy.

**Table 3:** Baseline confounders, *H. pylori* eradication regimens, and follow-up characteristics of included studies.

NO.	Study	Smoking (Exp/Ctr)	Alcohol(Exp/Ctr)	H. pylori Eradication (Exp only)	Follow-up Method (Both groups)	Follow-up Interval (Exp/Ctr)	Remarks
1	Yi-Chia Lee 2024 (18)	Current smokers: 4223/31497 (13.4%)/2984/31777 (9.4%)	≥2 times/week: 2102/31497 (6.7%)/1005/31777 (3.2%)	10-day sequential therapy; 10-day triple rescue; individualized regimens for 2nd-line failures; HPSA at 6–8 weeks (ITT: 91.9%, PP: 97.6%)	Outcome: Taiwan Cancer Registry + death database (coverage/accuracy >99%); telephone follow-up; colonoscopy for FIT+; cutoff Dec 2020, LTF ~0.2%.	Exp: q3d phone during treatment; 6–8w HPSA post-treatment; median FU 5.7y (4.9–6.5y) Ctr: median FU 5.4y (4.4–6.4y)	PP population
2	Kai-Feng Pan 2024 (8)	Former smokers: 8641/52026 (16.6%)/7990/50304 (15.9%)	Formerly drank: 10739/52026 (20.6%)/9875/50304 (19.6%)	10-day bismuth-containing quadruple therapy; ¹³C-UBT at 45 days, no re-treatment	Passive + active: Linqu Cancer Registry + China CDC; village doctor monthly records; 93.4% GC, 88.8% EC reviewed blindly.	Exp/Ctr: drug monitoring bid; 45d CUBT post-treatment; FU 11.8y	ITT population
3	Lingjun Yan 2022 (10)	Baseline daily smoking rate: 22.3%/22.3%	Baseline drinking rate: 22.4%/22.4%	2-week standard triple therapy; 1-week quadruple rescue; overall eradication 83.7%	Surveillance + prospective: Endoscopies 1999/2006/2020; regional cancer registry + Changle CDC; village doctor follow-up; 93.4% GC reviewed blindly.	Exp/Ctr: 6w ¹³C-UBT post-treatment; ¹³C-UBT q6mo; endoscopies 1999/2006/2020; FU 26.5y	ITT population
4	Wen-Qing Li 2019 (19)	Adjustment factor: Past smoking history/Past smoking history	Adjustment factor: Past alcohol consumption history/Not reported	2-week dual therapy; repeat same regimen for initial failures; placebo for blinding	Endoscopy + registry: Endoscopies 1999/2003; 6–12 monthly for dysplasia (2008–2017); village doctor records; GC confirmed via records.	Exp/Ctr: 3–6m ¹³C-UBT post-treatment; vitamin/garlic 7.3y; FU 22.3y (1995–2017)	ITT population
5	Zhou Liya 2014 (20)	Baseline: Smoking habits (no sig. diff.)/Not reported	Not reported/Not reported	1-week triple therapy; ¹³C-UBT at 1 month (eradication 88.89%); double-blind placebo control	Endoscopy + RUT: Follow-ups at 1/5/8/10y; biopsies per 1996 Sydney System; GC confirmed histologically.	Exp/Ctr: 1m ¹³C-UBT post-treatment; FU at 1/5/8/10y	ITT population
6	Tang 2010 (21)	Not reported/Not reported	Not reported/Not reported	Initial 7-day triple therapy; bismuth-containing quadruple rescue; overall eradication 82.1%	3y endoscopy: Hp confirmed by ¹³C-UBT/RUT/HE; endoscopy at 3y; GC confirmed via biopsy.	Exp/Ctr: 1m ¹³C-UBT post-treatment; FU 3y (2006–2009)	ITT population
7	W K Leung 2004 (22)	Not reported/Not reported	Not reported/Not reported	1-week triple therapy (OAC); RUT + histology confirmation (5-year eradication 74.5%)	Endoscopic monitoring: Endoscopies at baseline/5y (1y extra); updated Sydney System; outcomes via records; blind pathology.	Exp/Ctr: baseline endoscopy + Hp test; FU at 1/5y	ITT population
8	Benjamin C Y Wong 2012 (23)	Current smokers: 169/255 (70.7%)/166/258 (68.6%)	Current drinking: 158/255 (66.1%)/148/258 (61.2%)	7-day triple therapy; 2×2 factorial design; ¹³C-UBT at 45 days (ITT: 71.3%, PP: 78.2%); double-blind	Endoscopy + extended follow-up: Endoscopies 2004/2006; updated Sydney + Padova; 2007–2009 follow-up; blind review (3 pathologists, 95.9% agreement).	Exp/Ctr: 45d ¹³C-UBT post-treatment; 24m endoscopy; extended FU 2007–2009	ITT population

### 2.4 Quality assessment

We used RevMan 5.3 issued by the Cochrane Library to assess the risk of bias for eligible studies (17). We then evaluated the included studies using the GRADE approach. We classified the quality of evidence into high, moderate, and low grades according to risk of bias, imprecision, inconsistency, indirectness, and publication bias.

### 2.5 Statistical analysis

We hypothesized that the preventive effect of Hp eradication therapy on gastric cancer may vary according to study population recruitment strategies and sample sizes, and predefined corresponding subgroup criteria. The ultra-large sample community group included studies with a total sample size ≥ 50,000, which recruited participants via large-scale non-targeted community or public health platforms without prior high-risk screening; although two studies showed minor effect-size differences (RR = 0.86 vs. 0.95) due to different implementation pathways, all participants were from the general community, and the overall effect was significantly weaker than that in the conventional group. The conventional sample mixed group included studies with a total sample size < 50,000, which recruited via targeted enrollment of clinical high-risk individuals or small- to medium-sized community screening with prior risk assessment, and all were individual randomized controlled trials (iRCT). This subgroup classification aligns with clinical practice and evidence-based standards for Hp screening in China; the 50,000-sample threshold was chosen based on the typical scale of provincial and municipal community-wide Hp screening programs in China, and the classification was primarily based on sample size and recruitment logic, which directly reflect baseline gastric cancer risk and represent the key difference between the two groups. The criteria were jointly determined by the research team after literature screening and before statistical analysis, with no *post-hoc* adjustments, thus effectively reducing analysis bias.

Under the above subgroup analysis framework, we used RevMan 5.3 to pool the effect sizes of outcome measures. We took the intention-to-treat (ITT) population, person-years, sample size and follow-up duration as statistical indicators. We assessed heterogeneity by the χ² test and I² statistic. Significant heterogeneity was defined as P<0.10. Heterogeneity was classified as low (I² 0–40%), moderate (30–60%), high (50–90%) and very high (75–100%). We chose the pooled model based on heterogeneity results. The random-effects model was used when heterogeneity was significant, and the fixed-effects model was used when heterogeneity was not significant.

## 3 Results

### 3.1 Search results and information extraction

**Figure 1:**
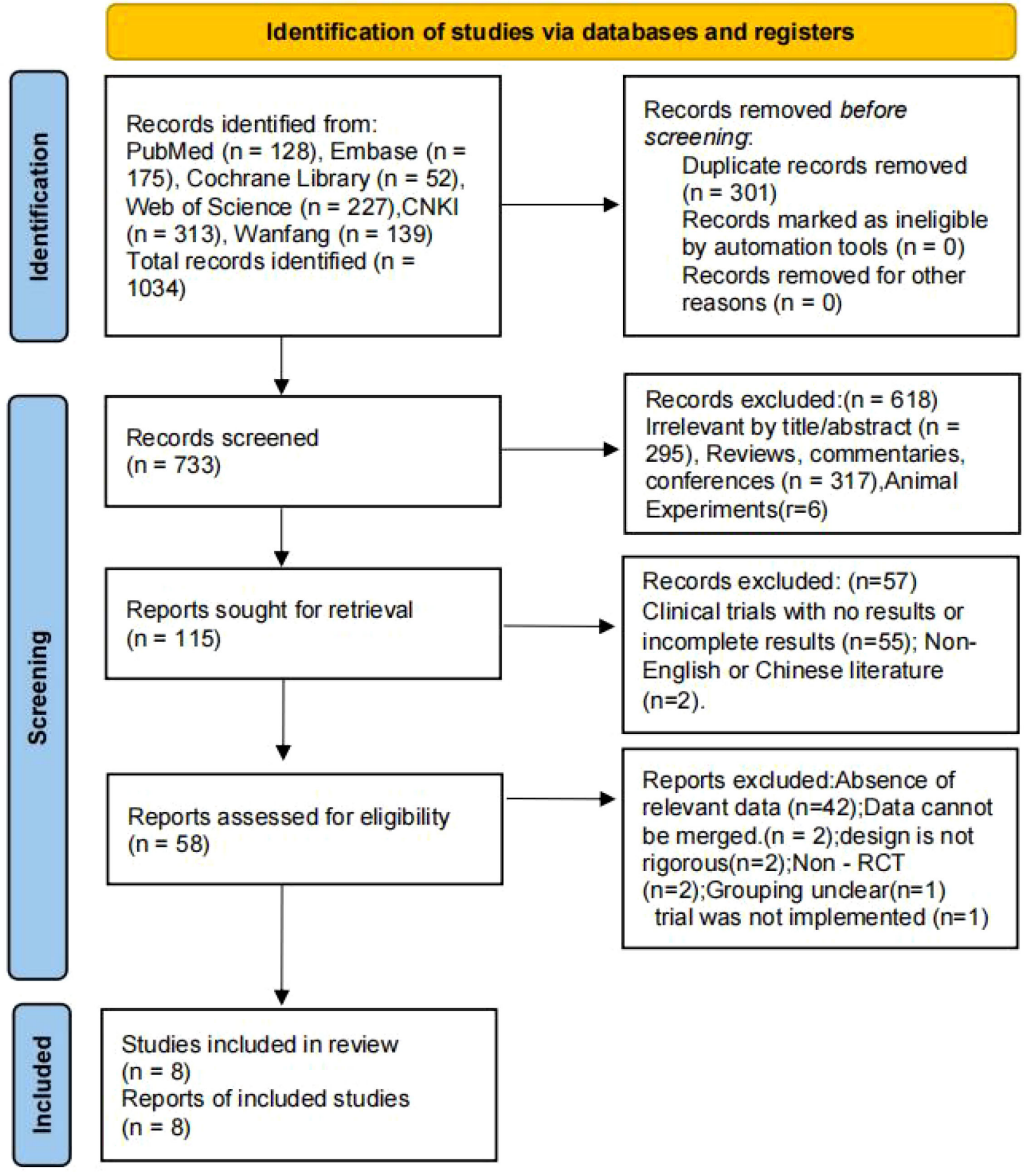
Search flow diagram.

A total of 1034 articles were initially identified. After comprehensive evaluation, 8 articles were finally included. The search process was presented as a PRISMA_2020 flow diagram (**Figure 1**). We extracted the basic characteristics of the included studies. The 8 studies were published between 2004 and 2024 and all were conducted in China. The total number of enrolled participants was 260609, and 118425 participants received Hp eradication therapy. All studies were individual randomized controlled trials except for Kai-Feng Pan et al., 2024 (8), which used cluster randomized controlled trials based on villages. The control types were different: 6 studies used placebo control, 1 used FIT screening alone as active control, and 1 used symptom-relief treatment as control. The study characteristics are shown in **Table 2**, **Table 3**, and the outcome indicators are shown in **Table 4**.

**Table 4:** Outcome measures.

No.	Study	Gastric cancer outcomes	
		Exp	Ctr
1	Yi-Chia Lee 2024 (18)	111/63508	163/88995
2	Kai-Feng Pan 2024 (8)	354/52026	399/50304
3	Lingjun Yan 2022 (10)	21/817	35/813
4	Wen-Qing Li 2019 (19)	40/1130	76/1128
5	Zhou Liya 2014 (20)	2/276	7/276
6	Tang 2010 (21)	1/118	4/118
7	W K Leung 2004 (22)	4/295	6/292
8	Benjamin C Y Wong 2012 (23)	3/255	1/258

### 3.2 Quality assessment

**Figure 2:**
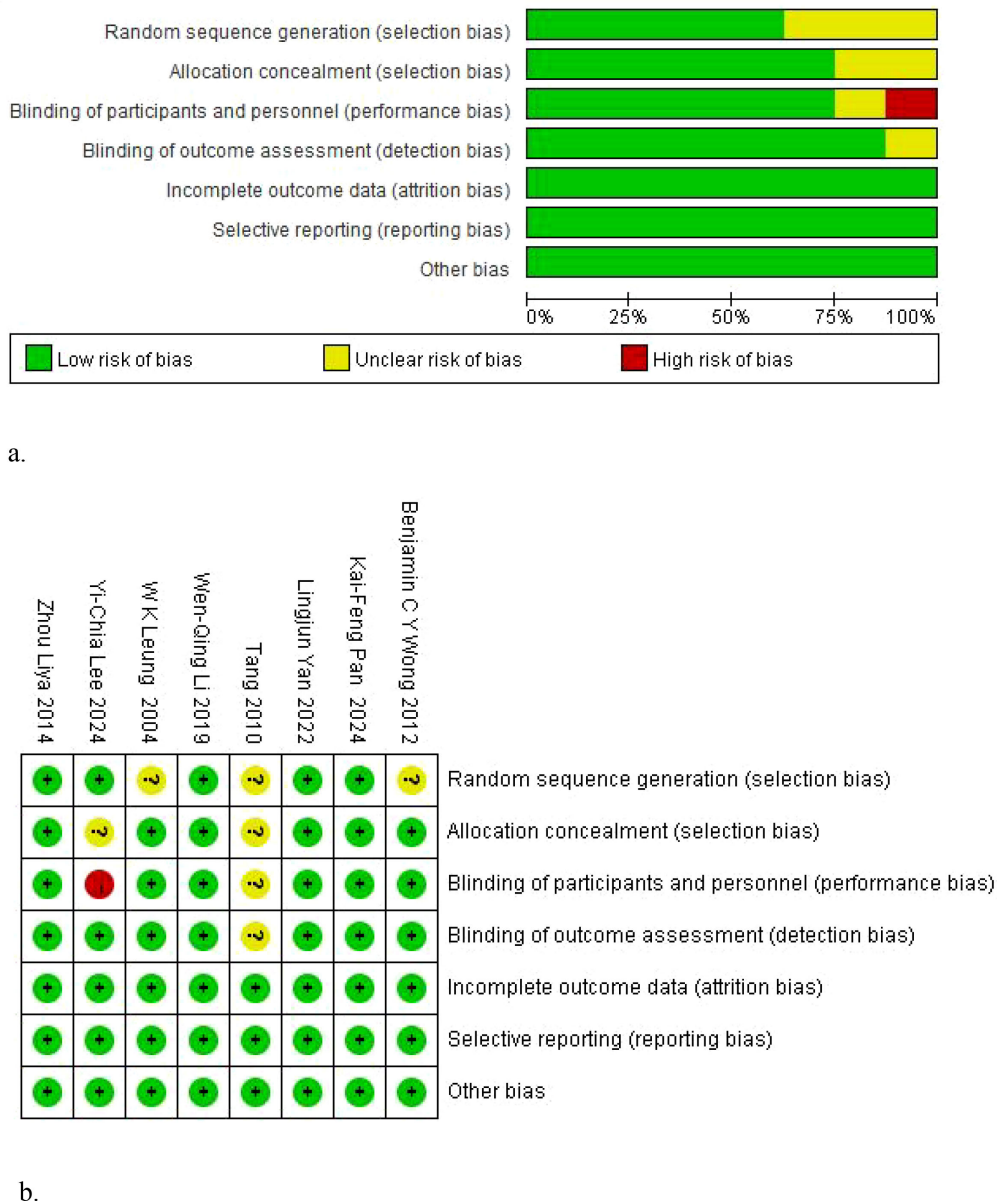
Risk of bias assessment. **(A)** Risk of bias graph. **(B)** Risk of bias summary.

A risk of bias assessment was conducted for the 8 included studies using RevMan 5.3. The results are shown in **Figure 2**.

#### 3.2.1 Random sequence generation and allocation concealment

Most studies clearly described the method of random sequence generation and were assessed as low risk. A few studies only mentioned random assignment without describing the specific method and were assessed as unclear risk. For allocation concealment, most studies described the implementation details and were defined as low risk, while other studies provided no relevant information and were defined as unclear risk.

#### 3.2.2 Blinding

In the implementation of blinding, the study by Tang et al. (21) showed unclear risk of bias in the dimensions of allocation concealment, blinding of participants and personnel, and blinding of outcome assessment because the details of blinding were not clearly described. The study by Yi-Chia Lee et al. (18) was rated as high risk of bias in this domain because blinding of participants and personnel could not be implemented due to its pragmatic design.

#### 3.2.3 Incomplete outcome data and selective reporting

All studies reported complete follow-up data with a low loss to follow-up rate, and the predefined primary outcome measures in the study protocols were all presented in the results. Therefore, both domains were assessed as low risk.

We further evaluated the 8 included studies using the GRADE quality assessment (**Table 5**). The initial quality of evidence for all studies was high. However, the overall evidence was finally downgraded to moderate due to limitations such as unclear or high risk in blinding implementation and insufficient reporting of allocation concealment in some studies. According to the risk of bias assessment table, the included studies performed well in random sequence generation, but there were still some unclear risks in blinding implementation and allocation concealment. This suggests that although the existing evidence can support the core conclusions, more high-quality studies with strict blinding and allocation concealment are still needed in the future to further verify the effect of Hp eradication therapy on the risk of gastric cancer.

**Table 5:** GRADE quality assessment.

Study	Initial Quality of Evidence	Limitations (Risk of Bias)	Inconsistency	Indirectness	Publication Bias	Final Quality of Evidence
Yi-Chia Lee 2024 (18)	High	Moderate	Moderate	Low	Low	Moderate
Kai-Feng Pan 2024 (8)	Moderate	Moderate	Low	Low	Low	Moderate
Lingjun Yan 2022 (10)	High	Moderate	Low	Low	Low	High
Wen-Qing Li 2019 (19)	High	Moderate	Low	Moderate	Low	Moderate
Zhou Liya 2014 (20)	High	Moderate	Low	Moderate	Low	Moderate
Tang 2010 (21)	High	Moderate	Low	Moderate	Unclear	Low
W K Leung 2004 (22)	High	Moderate	Low	Low	Low	High
Benjamin C Y Wong 2012 (23)	High	Low	Low	Moderate	Low	High

### 3.3 Publication bias assessment

**Figure 3:**
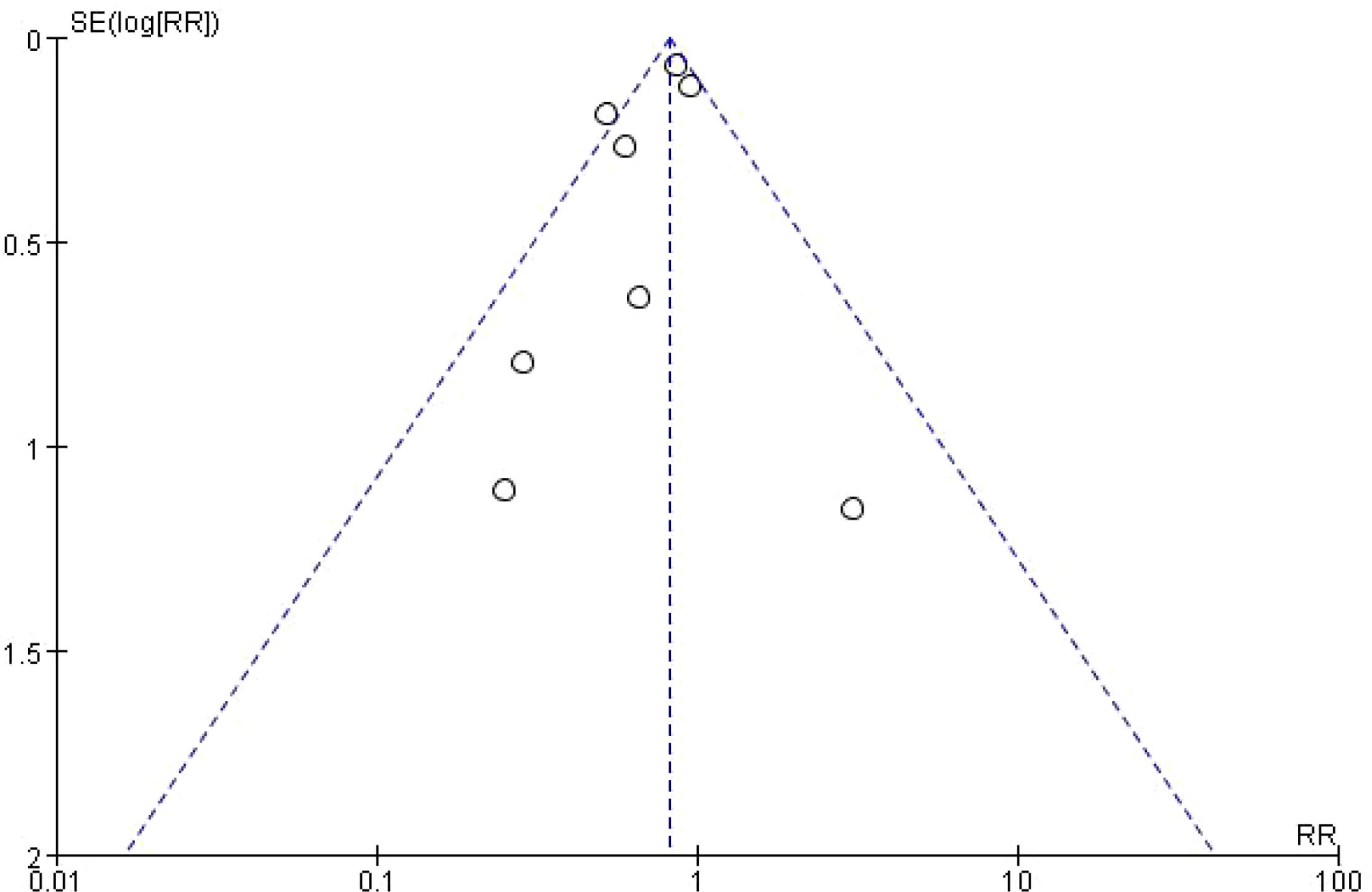
Funnel plot for assessment of publication bias.

This study used a funnel plot to qualitatively assess publication bias among the 8 included RCTs (**Figure 3**). The plot showed moderate asymmetry: overall, studies on the left side had more significant effect sizes (smaller RR values), while large-sample studies on the right side had milder effect sizes (closer to RR = 1). This phenomenon cannot be explained by publication bias alone; it is more likely related to multiple factors, including between-study heterogeneity (with sample size and recruitment strategy as core sources, I²=46%) leading to dispersed effect sizes, methodological flaws in some low-precision studies that may have amplified estimation bias, and the time-dependent nature of gastric cancer onset causing bidirectional fluctuations in short-term follow-up effect sizes. Despite this asymmetric distribution, the core conclusions of this study remain robust: the pooled analysis (RR = 0.82, 95% CI = 0.73-0.91, P = 0.0004) and stable results from large-sample and long-term follow-up subgroups (≥10 years: RR = 0.53, P<0.0001) both confirm that H. pylori eradication has clear value in preventing gastric cancer among targeted screening populations in China. Future studies should include gray literature, standardize research reports, and expand short-term follow-up sample sizes to further reduce the interference of heterogeneity and improve the authenticity of evidence.

### 3.4 Statistical analysis

**Figure 4:**
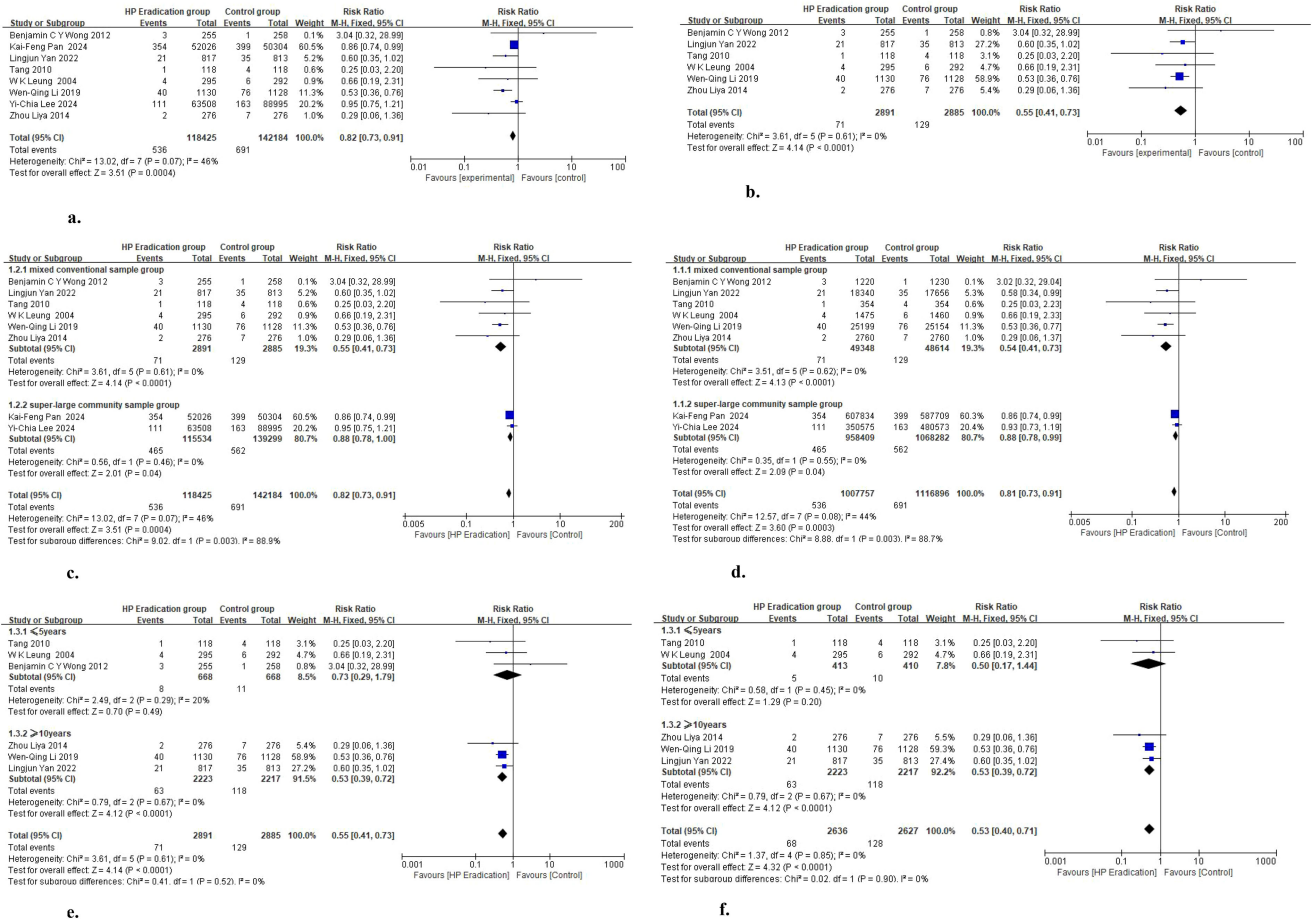
Meta-analysis forest plot. **(A)** Overall study Forest Plot Based on intention-to-treat population. **(B)** Subgroup analysis Forest Plot of ITT population after excluding 2 large-sample community studies. **(C)** Sample size subgroup analysis Forest Plot Based on ITT population. **(D)** Sample size subgroup analysis Forest Plot of ITT population based on person-years. **(E)** Follow-up duration subgroup analysis Forest Plot Based on ITT. **(F)** Follow-up duration subgroup analysis Forest Plot after excluding heterogeneous studies.

We performed a pooled analysis of the 8 included studies using the intention-to-treat (ITT) population (**Figure 4A**). The pooled effect size showed that I²=46%, suggesting low heterogeneity among the studies. We therefore used the fixed-effect model. The RR was 0.82, 95% CI = 0.73–0.91, P = 0.0004. These results confirmed that Hp eradication significantly reduced the risk of gastric cancer by 18%, and the outcome was highly statistically significant and reliable.

To verify the grouping logic and explore the source of heterogeneity, we performed a sensitivity analysis. After excluding the two ultra-large sample community studies by Kai-Feng Pan 2024 (8) and Yi-Chia Lee 2024 (18) (**Figure 4B**), the heterogeneity of the remaining six studies decreased from I²=46% to 0%. This result confirmed that sample size and recruitment strategy were the main sources of heterogeneity in this study.

Therefore, according to our predefined subgroup criteria, we classified the two studies by Kai-Feng Pan 2024 (8) and Yi-Chia Lee 2024 (18) into the ultra-large sample community group, and the remaining 6 small- and medium-sized studies recruited via targeted community screening or clinical settings into the conventional sample mixed group, then performed further subgroup analysis (**Figure 4C**). The results showed that the preventive effect of Hp eradication therapy against gastric cancer was RR = 0.88 (95% CI 0.78–1.00, I²=0%) in the ultra-large sample community group, while the effect was significantly stronger in the conventional sample mixed group (RR = 0.55, 95% CI 0.41–0.73, I²=0%). The significant between-subgroup difference (Chi²=9.02, P = 0.003) was the main source of the moderate heterogeneity (I²=46%, P = 0.07) in the overall analysis. The potential reason for this difference in effect size may be the different baseline gastric cancer risk and recruitment strategies between the two populations. The ultra-large sample community group mainly used unrestricted population-wide recruitment, which included many low-risk individuals and diluted the treatment benefit, with relatively limited screening intensity. In contrast, the conventional sample mixed group focused on clinically high-risk individuals with more precise intervention targets, which better reflected the value of eradication therapy in reversing precancerous lesions. This conclusion offers important guidance for public health strategies. It indicates that for the primary prevention of gastric cancer in China, universal Hp eradication strategies across entire communities should be used less frequently. Targeted Hp screening and standardized eradication should be prioritized in high-risk populations. Efficient utilization of medical resources can be achieved through risk stratification, which provides key evidence-based support for formulating precision-based and cost-effective strategies for gastric cancer prevention and control.

Based on the subgroup analysis by sample size in the ITT population that identified the source of heterogeneity, we further used person-years as the statistical basis to verify the above subgroups. We aimed to eliminate potential heterogeneity caused by follow-up time and adjust the effect of different follow-up durations across studies on the effect size. The results showed (**Figure 4D**) that the subgroup analysis based on person-years was not only consistent with the findings from the ITT population, but also reduced the overall heterogeneity to 44%. The I² value within both subgroups was 0%. This indicated that sample size remained the main source of overall heterogeneity after adjusting for follow-up time. It also confirmed that the cancer-preventive effect of Hp eradication therapy was stable in populations of different sizes, and only the effect strength differed according to sample characteristics.

After excluding the interference of ultra-large community populations on heterogeneity in the subgroup analysis by sample size based on person-years, we further explored the effect of follow-up time on the preventive effect of Helicobacter pylori eradication therapy against gastric cancer in the remaining studies. The results showed (**Figure 4E**) that gastric cancer risk was significantly reduced in the group with follow-up ≥ 10 years, with RR = 0.53 and P<0.0001, which confirmed the clear benefit of long-term follow-up. The group with follow-up ≤ 5 years had RR = 0.73, suggesting a downward trend in risk, but P = 0.49 and a wide confidence interval, showing no statistical significance. Meanwhile, the heterogeneity of I²=20% in this group indicated certain differences among studies. Further analysis found that the study by Benjamin C Y Wong et al. (23)had a wide confidence interval, which may affect the stability of the results. Therefore, we will separately exclude this study through sensitivity analysis to more accurately evaluate the cancer-preventive effect of Helicobacter pylori eradication therapy in short-term follow-up.

After excluding the 2012 study (**Figure 4F**), the I² value in the ≤5-year follow-up group decreased to 0, with RR = 0.50 and P = 0.20, and the result still did not reach statistical significance. This suggests that the potential cancer-preventive effect of short-term Helicobacter pylori eradication therapy is not statistically significant. The reason may be that the current sample size is insufficient to confirm this effect, and larger prospective randomized controlled trials are needed to further verify the potential cancer-preventive value of short-term eradication therapy. This result is also related to the pathophysiological characteristics of gastric cancer. The progression from chronic inflammation to precancerous lesions and then to gastric cancer in the gastric mucosa is a long-term process. The effects of mucosal injury repair and delayed lesion progression after eradication therapy are difficult to show as a significant decrease in gastric cancer incidence in the short term. It also indicates that short-term intervention cannot be used as a routine strategy for gastric cancer prevention and control. In clinical practice, Helicobacter pylori eradication intervention still needs to be combined with long-term follow-up.

## 4 Discussion

This systematic review and meta-analysis focused on Chinese Helicobacter pylori (Hp)-positive individuals with a follow-up duration of ≥3 years, and quantitatively evaluated the effect of Hp eradication therapy on gastric cancer risk. We verified the preventive effect of this therapy and its variation across different populations and follow-up periods, and identified the main sources of between-study heterogeneity. This study demonstrated that Hp eradication therapy reduced gastric cancer risk by 18% in Chinese Hp-positive individuals and by 45% in high-risk populations, findings highly consistent with numerous authoritative studies. Our results align with the pooled effect for East Asian populations reported in the Cochrane systematic review (24) (RR = 0.54, 46% risk reduction) and are strongly supported by a 2025 systematic review and meta-analysis published in Gastroenterology (25), which included 11 RCTs and 13 observational studies involving nearly 150,000 Hp-positive individuals and confirmed significant reductions in both gastric cancer risk (RR = 0.64, 95% CI 0.48–0.84) and related mortality (RR = 0.78, 95% CI 0.62–0.98) with evidence quality upgraded from low to moderate. Regionally, similar studies in Japan and South Korea showed 53% and 56% risk reductions, respectively, and the East Asian data from China, Japan, and South Korea included in our analysis confirmed cross-country consistency, providing robust evidence for clinical and public health decision-making. In contrast, relevant studies remain scarce in Western countries; only two non-East Asian studies (Colombia and the United States) included in the 2025 review and previous data demonstrated a weak, statistically non-significant preventive effect with no clear clinical value.

Pooled analysis showed that Hp eradication therapy significantly reduced gastric cancer risk by 18% (RR = 0.82, 95% CI = 0.73–0.91, P = 0.0004), confirming its clear preventive value in the Chinese population. Subgroup analysis further identified key effect differences: the medium- and small-scale community screening and clinical recruitment group showed a 45% risk reduction (RR = 0.55), which was significantly superior to the ultra-large sample community group (RR = 0.88). This difference may be attributed to population baseline risk: the medium- and small-scale group mainly recruited high-risk individuals with Hp-related symptoms or precancerous lesions via targeted screening, in whom eradication therapy exerted more pronounced effects on lesion reversal, whereas the ultra-large community group included a broad general population with low baseline gastric cancer risk, diluting the absolute treatment benefit. This aligns with the clinical principle that intervention benefits are more prominent in high-risk populations. For follow-up duration, the ≥10-year subgroup demonstrated a 47% risk reduction (RR = 0.53, P<0.0001) with stable and significant benefit, while the ≤5-year subgroup showed a risk-reduction trend (RR = 0.73) that did not reach statistical significance. After excluding heterogeneous studies, the effect size shifted toward stronger protection (RR = 0.50), indicating that the preventive effect of Hp eradication is time-dependent. This pattern is consistent with gastric cancer pathophysiology: the progression from chronic gastric inflammation and precancerous lesions to gastric cancer is a long-term process, and sufficient time is required for mucosal repair and delayed lesion progression after Hp eradication to produce a measurable reduction in cancer incidence, which short-term follow-up cannot adequately capture.

Moderate heterogeneity was observed in this study (I²=46%, P = 0.07), and two core sources of heterogeneity were identified through sensitivity analysis and subgroup analysis. Sample size and population type were the primary factor, and the effect size differed significantly between the ultra-large sample community group and the small- and medium-scale group (between-subgroup I²=90.2%, P = 0.001), with 0% heterogeneity within both groups, suggesting that population selection bias was the main driver of overall heterogeneity. This may be because large-scale community screening programs adopted cluster random sampling and enrolled general community populations with low baseline gastric cancer risk, leading to a low benefit of Hp eradication. In contrast, targeted screening enrolled high-risk populations recruited through clinical or small community screening, which were risk-enriched populations, and eradication therapy showed a more significant effect on reversing precancerous lesions. Meanwhile, most eradication regimens in the included trials were triple or quadruple therapies containing clarithromycin or metronidazole (such as OPZ+AMPC+CAM). In recent years, the resistance rates of clarithromycin and metronidazole have been increasing continuously in China, so the eradication rate of the historical regimens used in old trials will decrease significantly in current China. In addition, the eradication regimens in large-scale community programs were more uniform and difficult to adjust according to regional drug resistance, while clinical studies of targeted screening could adjust regimens individually, which may be a potential reason for the weaker effect of community programs and needs further trial data to verify the hypothesis. Follow-up time was another important source of heterogeneity. The effect size in the long-term follow-up (≥10 years) group was stable and significant, while the effect in the short-term group was unclear. This was related to the long-term latent characteristic of gastric cancer and also reflected the impact of inconsistent follow-up periods among different studies on the pooled results. Furthermore, subgroup analysis adjusted by person-years further verified the core role of sample size, with heterogeneity reduced to 44% and 0% within subgroups, indicating that after adjusting for follow-up time, differences in baseline population risk remained the core source of heterogeneity. Notably, heterogeneity in the short-term follow-up group decreased to 0% after excluding specific studies, suggesting that the follow-up quality or population characteristics of some studies may interfere with the assessment of short-term effects, and more high-quality studies are needed to verify the potential short-term benefits.

The quality of evidence from the 8 included high-quality randomized controlled trials was evaluated using the GRADE approach. Although the initial evidence grade was high, the overall quality was downgraded to moderate, mainly due to insufficient reporting of allocation concealment and defective or unclear blinding implementation. These biases did not alter the core finding that Hp eradication therapy reduces gastric cancer risk in the Chinese population, and only exerted a slight potential impact on the precision of the pooled effect size (RR = 0.82, 95%CI=0.73–0.91). Selection bias arose mainly from unclear allocation concealment in some studies, which could theoretically cause baseline imbalance and bidirectional bias; however, over 80% of studies had balanced key baseline characteristics, and subgroups were stratified by gastric cancer risk, so the influence of this bias was limited. Performance and detection biases stemmed from inadequate blinding, with some studies showing high risk or missing information on blinding. Unblinded participants and researchers tended to introduce performance bias and overestimate the protective effect, while unclear blinding of outcome assessment might interfere with gastric cancer endpoint evaluation. Overall, most biases in this study were moderate or unclear, with no widespread high-risk biases. The study had a low follow-up loss rate, with complete follow-up and outcome reporting. These issues only slightly widened the 95% confidence interval and marginally reduced effect size precision, but did not fundamentally change the core conclusion that Hp eradication therapy significantly reduces gastric cancer risk by 18% in Chinese Hp-positive individuals, nor did they affect the trend of subgroup analyses.

From clinical and public health perspectives, the findings of this study provide key evidence-based support for the primary prevention of gastric cancer. Hp eradication therapy serves as a core intervention for Chinese populations at high risk of gastric cancer, and its promotion should be prioritized in targeted small- and medium-scale community screening to maximize intervention benefits. Clinical decision-making should fully consider population characteristics and follow-up duration: for the general community population, screening and intervention should be implemented with risk stratification, while long-term follow-up is still necessary for high-risk populations to achieve cancer prevention, even if no statistically significant short-term benefit is observed. At the public health level, Hp infection prevention and control strategies can be optimized based on this study, with eradication therapy prioritized in regions and populations with a high incidence of gastric cancer to reduce the population-level burden of the disease. Meanwhile, patients should be fully informed of the long-term benefits of the treatment to improve treatment adherence.

This study has several limitations. First, the overall evidence quality of the included studies was moderate, mainly owing to insufficient reporting of allocation concealment and flawed or unclear blinding implementation (e.g., blinding of participants and researchers was unfeasible in pragmatic community screening designs); these biases did not alter the core effect direction or subgroup analysis trends, but introduced minor performance and detection biases, slightly reducing the precision of pooled risk estimates and failing to fully exclude small deviations between the pooled RR value and the true effect. Second, although sample size and follow-up duration were identified as the two core sources of heterogeneity, residual heterogeneity may be affected by unmeasured confounders such as eradication regimens, eradication rates, smoking, and alcohol consumption, which were not included in subgroup analyses and may compromise result accuracy. Third, the potential benefits of short-term follow-up (≤5 years) lack sufficient sample validation among Chinese populations due to the limited number of high-quality studies; qualitative funnel plot assessment indicated a potential risk of publication bias (moderate asymmetric distribution) related to between-study heterogeneity, methodological flaws in some studies, and the time-dependent nature of gastric cancer, yet this bias was not verified quantitatively, rendering the true magnitude of short-term effects unclear. Fourth, subgroup analyses by age, gender, or lesion severity were not performed for the included studies, so the moderating effects of these factors on treatment outcomes cannot be determined, and comparative data on the efficacy of different eradication regimens are lacking. Fifth, indicators including gastric cancer-related epidemiological models and CAG-a definitions were not incorporated into the inclusion/exclusion criteria or subgroup analyses; given the close association between CAG-a antibodies and gastric cancer carcinogenesis and the availability of relevant data in some included studies, this omission may increase residual heterogeneity and reduce result accuracy. Furthermore, this study focused solely on Chinese populations, whose Hp infection rate, strain subtypes, dietary and clinical management patterns, and baseline gastric cancer risk differ significantly from those in Western countries and low-incidence regions, limiting the external validity of the conclusions. Future studies should conduct targeted validation based on regional epidemiological characteristics, actively include gray literature to reduce the impact of publication bias, and improve the comprehensiveness and reliability of the evidence.

Future research directions should focus on the following aspects: first, large-sample and methodologically rigorous randomized controlled trials with standardized random sequence generation, allocation concealment, and blinding designs should be implemented to improve the quality of evidence; second, individualized studies of Hp eradication therapy should be promoted to explore predictive factors of treatment efficacy, including age, baseline lesion status, and lifestyle, to achieve precise intervention; third, the sample size of short-term follow-up studies should be expanded to clarify the potential benefits and applicable populations of short-term treatment; fourth, study reporting should be standardized, with detailed documentation of parameters such as treatment regimens, eradication rates, and follow-up quality to reduce heterogeneity; fifth, long-term follow-up studies should be performed to verify the long-term safety and durable cancer-preventive effect of eradication therapy, providing a more comprehensive basis for the optimization of public health strategies.

## 5 Conclusion

Based on evidence from randomized controlled trials of Helicobacter pylori (Hp)-positive Chinese people with follow-up of ≥3 years, Hp eradication therapy can significantly reduce the risk of gastric cancer. The benefit is more obvious in people recruited through small- and medium-scale community screening and clinical settings. The cancer-preventive effect is clear with long-term follow-up of ≥10 years. We recommend promoting this therapy in clinically high-risk populations and precise community screening to improve the effect of primary prevention of gastric cancer in China.

## Data Availability

The original contributions presented in the study are included in the article/**Supplementary Material**. Further inquiries can be directed to the corresponding author.
