# Supplementary material for: Gastric cancer prevention by H. pylori eradication in China: a meta-analysis of 8 high-quality RCTs in targeted screening populations
Source: Front Oncol. 2026 Apr 1;16:1789299. doi: 10.3389/fonc.2026.1789299 (PMC13079041; doi:10.3389/fonc.2026.1789299)

MeSH descriptor: [Stomach Neoplasms] explode all trees

(Stomach Neoplasm):ti,ab,kw OR (Gastric Neoplasms):ti,ab,kw OR (Gastric Neoplasm):ti,ab,kw OR (Neoplasm, Gastric):ti,ab,kw OR (Neoplasms, Gastric):ti,ab,kw OR (Neoplasms, Stomach):ti,ab,kw OR (Cancer of Stomach):ti,ab,kw OR (Stomach Cancers):ti,ab,kw OR (Cancer of the Stomach):ti,ab,kw OR (Gastric Cancer):ti,ab,kw OR (Cancer, Gastric):ti,ab,kw OR (Cancers, Gastric):ti,ab,kw OR (Gastric Cancers):ti,ab,kw OR (Stomach Cancer):ti,ab,kw OR (Cancers, Stomach):ti,ab,kw OR (Cancer, Stomach):ti,ab,kw OR (Gastric Cancer, Familial Diffuse):ti,ab,kw

MeSH descriptor: [Helicobacter pylori] explode all trees

(Campylobacter pyloridis):ti,ab,kw OR (Campylobacter pylori):ti,ab,kw OR (Helicobacter nemestrinae):ti,ab,kw

MeSH descriptor: [Disease Eradication] explode all trees

(Eradication, Disease):ti,ab,kw OR (Eradications, Disease):ti,ab,kw OR (Disease Elimination):ti,ab,kw OR (Disease Eliminations):ti,ab,kw OR (Elimination, Disease):ti,ab,kw OR (Eliminations, Disease):ti,ab,kw

(#1 or #2) and ((#3 or #4) and (#5 or #6))


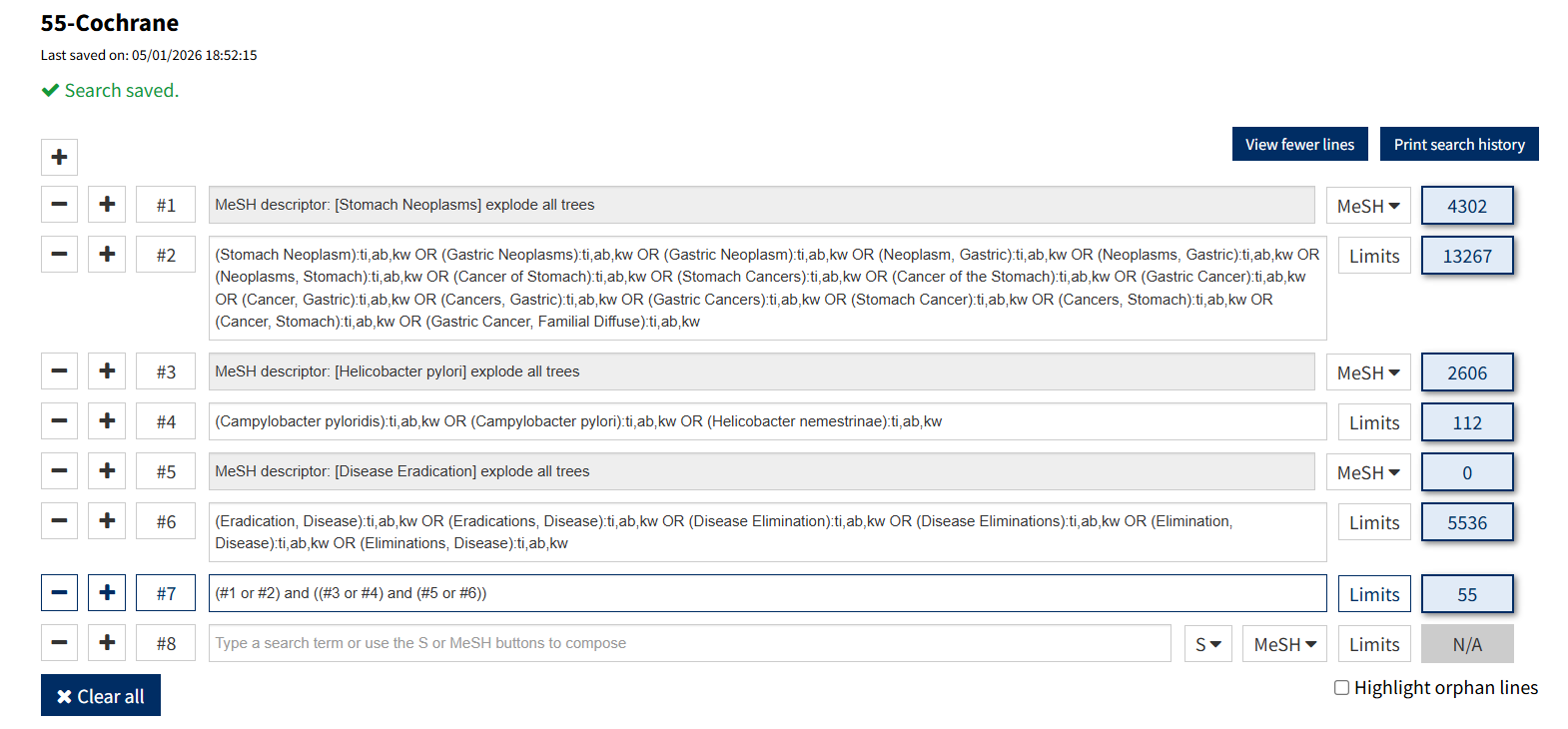

Supplement: Supplementary file 1 [file DataSheet1.zip › Supplement Files/Supplement File1/Cochrane search strategy.docx]
