# Supplementary material for: Gastric cancer prevention by H. pylori eradication in China: a meta-analysis of 8 high-quality RCTs in targeted screening populations
Source: Front Oncol. 2026 Apr 1;16:1789299. doi: 10.3389/fonc.2026.1789299 (PMC13079041; doi:10.3389/fonc.2026.1789299)
Supplement: Supplementary file 1 [file DataSheet1.zip › Supplement Files/Supplement File1/Embase search strategy.docx]

Embase search strategy

#1

'stomach tumor'/exp OR 'stomach tumor' OR 'neoplasm, stomach':ab,ti OR 'stomach neoplasm':ab,ti OR 'gastric neoplasms':ab,ti OR 'gastric neoplasm':ab,ti OR 'neoplasm, gastric':ab,ti OR 'neoplasms, gastric':ab,ti OR 'neoplasms, stomach':ab,ti OR 'cancer of stomach':ab,ti OR 'stomach cancers':ab,ti OR 'cancer of the stomach':ab,ti OR 'gastric cancer':ab,ti OR 'cancer, gastric':ab,ti OR 'cancers, gastric':ab,ti OR 'gastric cancers':ab,ti OR 'stomach cancer':ab,ti OR 'cancers, stomach':ab,ti OR 'cancer, stomach':ab,ti OR 'gastric cancer, familial diffuse':ab,ti

#2

(((('helicobacter pylori'/exp OR campylobacter) AND subsp. AND pylori OR campylobacter) AND pyloridis OR campylobacter) AND pylori OR helicobacter) AND nemestrinae OR hp

#3

'randomized controlled trial'/exp OR 'rct':ab,ti OR 'randomized':ab,ti OR 'cohort':ab,ti OR 'placebo':ab,ti

#4

'eradication':ab,ti OR 'eradication therapy':ab,ti

#5

#2 AND #4

#6

#1 AND #3 AND #5
