# Supplementary material for: Gastric cancer prevention by H. pylori eradication in China: a meta-analysis of 8 high-quality RCTs in targeted screening populations
Source: Front Oncol. 2026 Apr 1;16:1789299. doi: 10.3389/fonc.2026.1789299 (PMC13079041; doi:10.3389/fonc.2026.1789299)
Supplement: Supplementary file 1 [file DataSheet1.zip › Supplement Files/Supplement File1/PubMed search strategy.docx]

| #1 | (Stomach Neoplasms [Mesh]) OR (Neoplasm, Stomach[Title/Abstract] OR Stomach Neoplasm[Title/Abstract] OR Gastric Neoplasms[Title/Abstract] OR Gastric Neoplasm[Title/Abstract] OR Neoplasm, Gastric[Title/Abstract] OR Neoplasms, Gastric[Title/Abstract] OR Neoplasms, Stomach[Title/Abstract] OR Cancer of Stomach[Title/Abstract] OR Stomach Cancers[Title/Abstract] OR Cancer of the Stomach[Title/Abstract] OR Gastric Cancer[Title/Abstract] OR Cancer, Gastric[Title/Abstract] OR Cancers, Gastric[Title/Abstract] OR Gastric Cancers[Title/Abstract] OR Stomach Cancer[Title/Abstract] OR Cancers, Stomach[Title/Abstract] OR Cancer, Stomach[Title/Abstract] OR Gastric Cancer, Familial Diffuse[Title/Abstract]) |
| --- | --- |
| #2 | (Helicobacter pylori [Mesh]) OR (Campylobacter pylori subsp. pylori[Title/Abstract] OR Campylobacter pyloridis[Title/Abstract] OR Campylobacter pylori[Title/Abstract] OR Helicobacter nemestrinae[Title/Abstract] OR HP[Title/Abstract]) |
| #3 | ((Disease Eradication [Mesh]) ) OR (Disease Eradications[Title/Abstract] OR Eradication, Disease[Title/Abstract] OR Eradications, Disease[Title/Abstract] OR Disease Elimination[Title/Abstract] OR Disease Eliminations[Title/Abstract] OR Elimination, Disease[Title/Abstract] OR Eliminations, Disease[Title/Abstract] OR Elimination*[Title/Abstract] OR Eradication*[Title/Abstract]) |
| #4 | ((((((Randomized Controlled Trials as Topic[Mesh]) OR (Randomized controlled trial[Title/Abstract])) OR (Clinical Trials, Randomized[Title/Abstract])) OR (Trials, Randomized Clinical[Title/Abstract])) OR (Controlled Clinical Trials, Randomized[Title/Abstract])) OR (RCT[Title/Abstract])) OR (Randomized[Title/Abstract]) |
| #5 | #1AND#2AND#3AND#4 |
