# Supplementary material for: Gastric cancer prevention by H. pylori eradication in China: a meta-analysis of 8 high-quality RCTs in targeted screening populations
Source: Front Oncol. 2026 Apr 1;16:1789299. doi: 10.3389/fonc.2026.1789299 (PMC13079041; doi:10.3389/fonc.2026.1789299)
Supplement: Supplementary file 1 [file DataSheet1.zip › Supplement Files/Supplement File1/Wanfang search strategy.docx]

主题:(胃癌) and 主题:(根除幽门螺杆菌) and 主题:(随机对照)
