# Supplementary material for: Gastric cancer prevention by H. pylori eradication in China: a meta-analysis of 8 high-quality RCTs in targeted screening populations
Source: Front Oncol. 2026 Apr 1;16:1789299. doi: 10.3389/fonc.2026.1789299 (PMC13079041; doi:10.3389/fonc.2026.1789299)
Supplement: Supplementary file 1 [file DataSheet1.zip › Supplement Files/Supplement File1/Wos search strategy.docx]

# Searches:

1: TS=(Stomach Neoplasms OR Neoplasm, Stomach OR Stomach Neoplasm OR Gastric Neoplasms OR Gastric Neoplasm OR Neoplasm, Gastric OR Neoplasms, Gastric OR Neoplasms, Stomach OR Cancer of Stomach OR Stomach Cancers OR Cancer of the Stomach OR Gastric Cancer OR Cancer, Gastric OR Cancers, Gastric OR Gastric Cancers OR Stomach Cancer OR Cancers, Stomach OR Cancer, Stomach OR Gastric Cancer, Familial Diffuse)

2: TS=(Helicobacter pylori OR Campylobacter pylori subsp. pylori OR Campylobacter pyloridis OR Campylobacter pylori OR Helicobacter nemestrinae OR HP)

3: TS=(Disease Eradication OR Disease Eradications OR Eradication, Disease OR Eradications, Disease OR Disease Elimination OR Disease Eliminations OR Elimination, Disease OR Eliminations, Disease OR Eradication* OR Elimination*)

4: TS=(Randomized Controlled Trial OR RCT OR randomised OR placebo OR random)

5: #2 AND #3

6: #1 AND #5 AND #4
