# Supplementary material for: Gastric cancer prevention by H. pylori eradication in China: a meta-analysis of 8 high-quality RCTs in targeted screening populations
Source: Front Oncol. 2026 Apr 1;16:1789299. doi: 10.3389/fonc.2026.1789299 (PMC13079041; doi:10.3389/fonc.2026.1789299)
Supplement: Supplementary file 1 [file DataSheet1.zip › Supplement Files/Supplement File2/PRISMA_2020_flow_diagram_new_SRs_v1.docx]

**Identification of studies via databases and registers**

Records removed *before screening*:

Duplicate records removed (n = 301)

Records marked as ineligible by automation tools (n = 0)

Records removed for other reasons (n = 0)

Records identified from:

PubMed (n = 128), Embase (n = 175), Cochrane Library (n = 52), Web of Science (n = 227),CNKI (n = 313), Wanfang (n = 139)

Total records identified (n = 1034)

**Identification**

Records excluded:(n = 618)

Irrelevant by title/abstract (n = 295), Reviews, commentaries, conferences (n = 317),Animal Experiments(r=6)

Records screened

(n = 733)

Records excluded: (n=57) Clinical trials with no results or incomplete results (n=55); Non-English or Chinese literature (n=2).

Reports sought for retrieval

(n = 115)

**Screening**

Reports excluded:Absence of relevant data (n=42);Data cannot be merged.(n = 2);design is not rigorous(n=2);Non - RCT (n=2);Grouping unclear(n=1)

trial was not implemented (n=1)

Reports assessed for eligibility

(n = 58)

Studies included in review

(n = 8)

Reports of included studies

(n = 8)

**Included**

*Consider, if feasible to do so, reporting the number of records identified from each database or register searched (rather than the total number across all databases/registers).

**If automation tools were used, indicate how many records were excluded by a human and how many were excluded by automation tools.

Source: Page MJ, et al. BMJ 2021;372:n71. doi: 10.1136/bmj.n71.

This work is licensed under CC BY 4.0. To view a copy of this license, visit <https://creativecommons.org/licenses/by/4.0/>
